# Identification of Matrine as a Novel Regulator of the CXCR4 Signaling Axis in Tumor Cells

**DOI:** 10.3390/ijms21134731

**Published:** 2020-07-02

**Authors:** Young Yun Jung, Jae-Young Um, Acharan S. Narula, Ojas A. Namjoshi, Bruce E. Blough, Alan Prem Kumar, Kwang Seok Ahn

**Affiliations:** 1 Department of Science in Korean Medicine, Kyung Hee University, 24 Kyungheedae-ro, Dongdaemun-gu, Seoul 02447, Korea; ve449@naver.com (Y.Y.J.); jyum@khu.ac.kr (J.-Y.U.); 2 Narula Research, Chapel Hill, NC 27516, USA; anarula1@nc.rr.com; 3 Center for Drug Discovery, RTI International, Research Triangle Park, Durham, NC 27709, USA; onamjoshi@rti.org (O.A.N.); beb@rti.org (B.E.B.); 4 Cancer Science Institute of Singapore, National University of Singapore, Singapore 117599, Singapore; 5 Department of Pharmacology, Yong Loo Lin School of Medicine, National University of Singapore, Singapore 117600, Singapore

**Keywords:** matrine, CXCR4, NF-ĸB, MMP-9/2

## Abstract

Matrine, a quinolizidine alkaloid, is commonly employed for treating various viral and inflammatory disorders. Here, we have evaluated matrine for its activity on C-X-C chemokine receptor type 4 (CXCR4) and matrix metalloproteinases (MMP-9/2) expression, and its potential to affect tumor metastasis and invasion. The effects of matrine on CXCR4, MMP-9/2, and nuclear factor κB (NF-κB) activation in lung (A549), prostate (DU145), and pancreas (MIA PaCa-2) cells were investigated by diverse techniques. The expression level of CXCR4 and MMP-9/2 was analyzed by western blot analysis and reverse transcription polymerase chain reaction. NF-κB activation was also evaluated by western blot analysis, electrophoretic mobility shift assay as well as immunocytochemical experiments. Furthermore, we monitored cell invasion and metastasis activities by wound healing and Boyden chamber assays. We noted that matrine induced a down-regulation of CXCR4 and MMP-9/2 at both protein and mRNA levels. In addition, matrine negatively regulated human epidermal growth factor receptor 2 (HER2) and C-X-C Motif Chemokine Ligand 12 (CXCL12)-induced CXCR4 expression. Moreover, NF-κB suppression by matrine led to inhibition of metastatic potential of tumor cells. Our results suggest that matrine can block the cancer metastasis through the negative regulation of CXCR4 and MMP-9/2 and consequently it can be considered as a potential candidate for cancer therapy.

## 1. Introduction

Metastasis consists of complex and diverse process that facilitates the expansion of tumor cells from their original site to other organs [1]. As a result of such complexities, metastasis has been considered as a major cause of mortality and morbidity in most cancers [2]. Typically, 57% of patients with lung cancer have metastatic disease and the survival rate is only 5%, which is significantly lower than the survival rate of around 57% for patients with localized stage disease [3]. In patients with prostate cancer, it was reported that the survival rate of patients with local metastasis was higher than that of patients with multiple metastatic lesions [4,5]. In addition, patients with pancreatic cancer, survival rate decreased from 20.2% to 0% as the degree of metastasis to lymph nodes increased [6]. The metastatic process consists of a series of consecutive linked steps, including embolization, invasion, survival in the circulation, and migration to various other organs [7]. The chemokines family proteins can regulate multiple processes such as adhesion, hematopoiesis, and angiogenesis and mediate the metastasis [8]. These chemokines can exert their actions on leukocytes through selective membrane-G protein binding receptors and can be divided into four subgroups, CC, C, CX3C, and CXC [9,10].

CXCR4, the receptor of the stromal cell-derived factor-1α (SDF-1α, also known as CXCL12) is one of the chemokines that has been researched extensively for its involvement in cancer metastasis and migration [11]. CXCL12 (SDF-1), ligand of CXCR4, is expressed at the sites of tumor metastasis and is involved in homing of the tumors to different organs [12]. CXCR4 is constitutively expressed in various cancers including lung [13], pancreatic [14], breast [15,16], prostate [17,18], and ovarian tumor cells [19]. It has been reported that CXCR4 can regulate vascularization in gastrointestinal tract [17], migration of germ cells [20], and infection of host cells with HIV [21]. Thus, CXCR4 can be developed as a therapeutic target for regulating metastasis [22,23,24].

Interestingly, HER2 receptor tyrosine kinase can also cause an upregulation of CXCR4 expression that can mediate breast cancer invasion [25]. Additionally, it has been demonstrated that CXCR4 inhibitors (AMD3100 and YN14003) can significantly mitigate tumor progression in HER2 overexpressing breast tumor models thereby indicating that CXCR4 attenuation could be a useful strategy for targeting HER2 breast cancer patients [26]. Furthermore, metastasis of malignant cells can result in rapid degradation of stromal extracellular matrix (ECM) and basement membrane [27]. This process can be mediated by various inflammatory enzymes such as MMP-9 and MMP-2 that can promote degradation of diverse ECM components [28,29,30,31,32].

Natural agents have the potential to target various hallmarks of cancer cells, including invasion and metastasis [23,33,34,35,36,37,38,39,40,41,42,43,44,45,46,47,48,49]. Matrine (C_15_H_24_N_2_O) is a quinolizidine alkaloid found in *Sophora flavescens,* that has been used in traditional Chinese herb medicines. In addition, matrine can be applied against various diseases due to its potent anti-viral and anti-inflammatory effects [50,51]. In previous studies, matrine was reported to reduce cellular growth and invasion potential of CRPC cells via suppression of MMP-9 and MMP-2 activities [52,53,54]. Because, upregulation of CXCR4 can affect the expression and activity of MMPs and consequently promote cell invasion and migration, [55], we analyzed here the impact of matrine on both CXCR4 as well as MMPs expression.

In our study, we focused on actions of matrine upon CXCR4 and MMPs activities in different cancer cell lines A549, DU145, and MIA PaCa-2, which display high basal expression of CXCR4 and MMPs. We noticed a substantial downregulation of CXCL12-induced CXCR4 expression by matrine. Consequently, this alkaloid exhibited anti-metastasis activities via affecting the CXCR4 and MMPs levels in human lung, prostate, and pancreatic cancer cells.

## 2. Results

### 2.1. Matrine Suppresses the CXCR4 Expression in A549, DU145, and MIA PaCa-2 Cells

**Figure 1:**
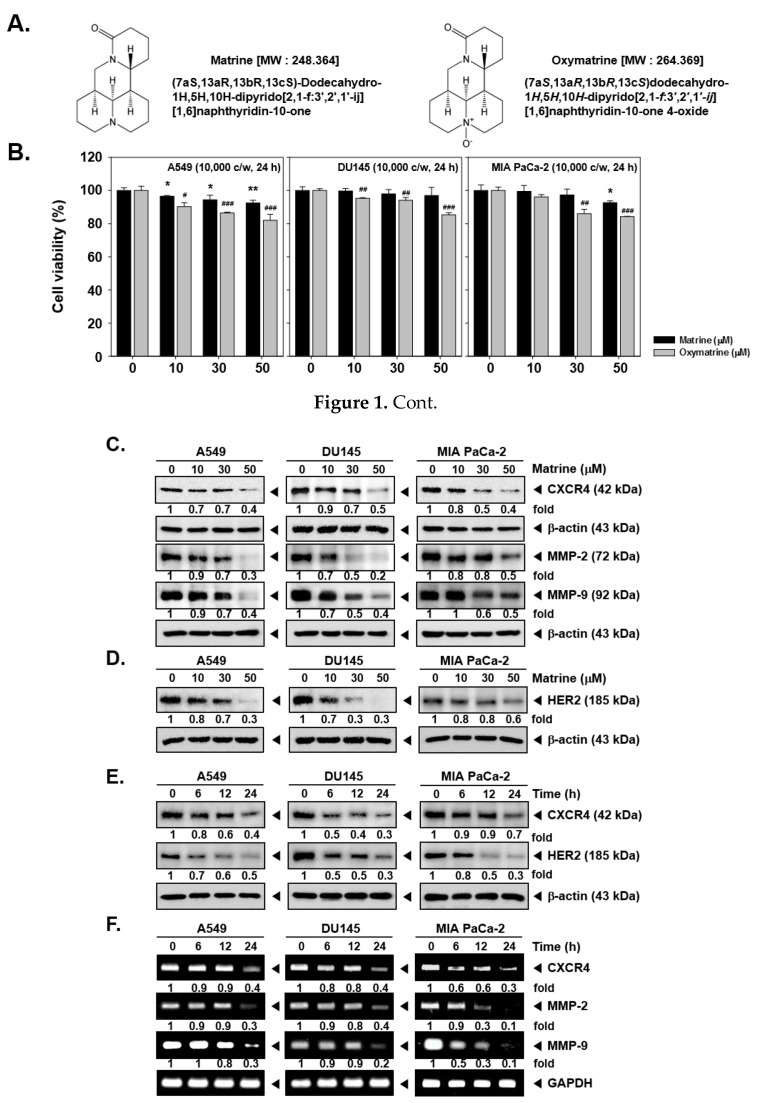
Effects of matrine on CXCR4 levels on A549, DU145 and MIA PaCa-2 cells. (**A**) Chemical structure of matrine. (**B**) A549, DU145 and MIA PaCa-2 cells (1 × 10^4^ cells/well) were treated with matrine for 24 h. Then cell viability was measured by MTT assay. (**C** and **D**) A549, DU145 and MIA PaCa-2 cells (5 × 10^5^ cells/well) were treated with matrine for 24 h in 37 °C, 5% CO_2_ incubator. Cells were harvested and whole cell lysates were prepared for western blot analysis. Proteins were separated on SDS-PAGE gels and transferred to nitrocellulose membranes. Membranes were probed with anti-CXCR4, anti-MMP-2, anti-MMP-9, and anti-HER2 antibodies. The same membranes were stripped and probed with β-actin antibodies. (**E**) A549, DU145, and MIA PaCa-2 cells (5 × 10^5^ cells/well) were treated with 50 µM of matrine for indicated time intervals in 37 °C, 5% CO_2_ incubator. Whole cell lysates were prepared for western blot analysis then probed with anti-CXCR4, and anti-HER2 antibodies. Same membranes were stripped to confirm the β-actin levels. (**F**) A549, DU145 and MIA PaCa-2 cells (5 × 10^5^ cells/well) were treated with 50 µM of matrine for several time intervals. mRNA level was measured by RT-PCR. We have used GAPDH as a loading control. Then expression levels of CXCR4, MMP-2, MMP-9 was compared on each cell lines. All experiments were performed independently at least 3 times and representative data are shown. ^**^ *p* < 0.01, ^*^ *p* < 0.05 vs. non-treated (NT) cells with matrine and ^###^ *p* < 0.001, ^##^ *p* < 0.01, ^#^ *p* < 0.05 vs. non-treated (NT) cells with oxymatrine.

To decipher the action of matrine on CXCR4 expression, the cell viability was first confirmed by MTT assay. A549, DU145, and MIA PaCa-2 cells were treated with matrine or oxymatrine (at doses of 0, 10, 30, 50 µM) for 24h (Figure 1B).

The results showed that 50 µM of matrine treated cells had more than 80% of cell viability and thus matrine exhibited low cytotoxicity against A549, DU145, and MIA PaCa-2 cells. On the other hand, since oxymatrine exhibited higher cytotoxicity, we decided to study the effects of matrine, which showed comparatively lower cytotoxicity for additional experiments.

Then we observed the expression of CXCR4 levels by western blot analysis and RT-PCR. As shown in Figure 1C, matrine decreased the CXCR4 expression as well as MMP-2 and MMP-9 levels in A549, DU145, and MIA PaCa-2 cells. In particular, matrine-induced suppression in MMP-9 and MMP-2 levels was more clearly observed in A549 and DU145 cells. Matrine also attenuated the HER2 expression in these cells depending on the concentrations employed (Figure 1D). It showed a similar pattern in a time dependent treatment of matrine, as shown in Figure 1E, CXCR4 and HER2 expression was suppressed in time dependent fashion.

### 2.2. Matrine Down-Regulates MMP-2 and MMP-9 Expression

Then we confirmed whether matrine can also affect CXCR4, MMP-2, and MMP-9 mRNA levels in tumor cells. A time dependent matrine treatment led to a marked reduction in CXCR4, MMP-2, and MMP-9 mRNA in the indicated cell lines (Figure 1F).

### 2.3. Matrine Attenuates Migration Activity in A549, DU145, and MIA PaCa-2 Cells

**Figure 2:**
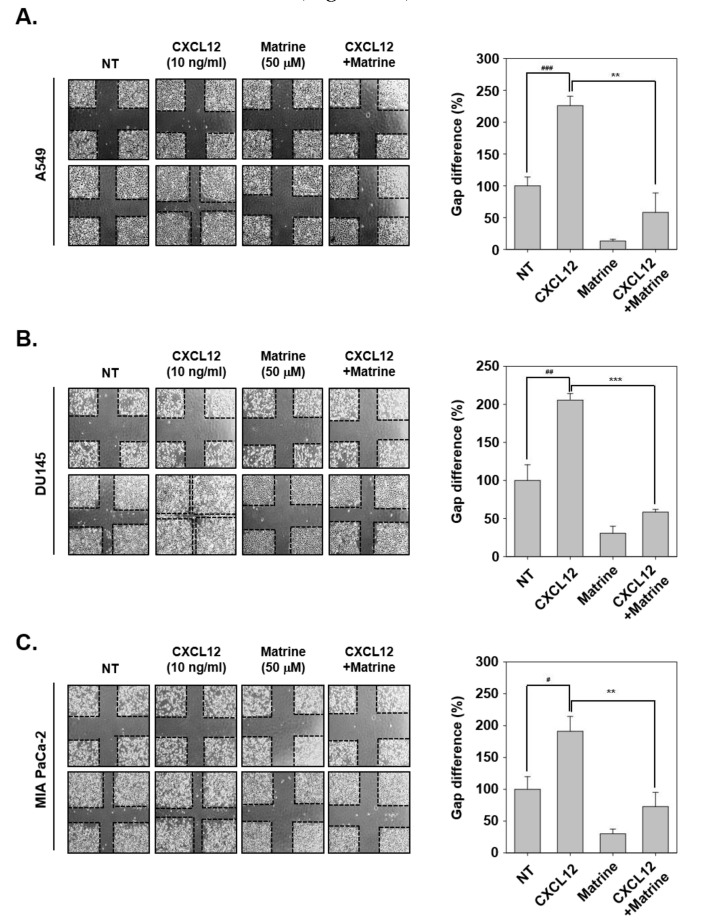
Matrine inhibits the cell migration on A549, DU145, and MIA PaCa-2 cells. (**A**–**C**) A549, DU145, and MIA PaCa-2 cells (5 × 10^5^ cells/well) were seeded on 6 well plate. Wound healing assay was performed on each well, cells were scarred then CXCL12 (10 ng/mL) and matrine (50 µM) were treated in serum-free media for 24 h. We observed conditions at 0 h, and compared with cell conditions at 24 h. All experiments were performed independently at least three times and representative data are shown. ^###^ *p* < 0.001, ^##^ *p* < 0.01, ^#^ *p* < 0.05 vs. non-treated (NT) cells and ^***^ *p* < 0.001, ^**^ *p* < 0.01 vs. CXCL12 + matrine treated cells.

Next, we evaluated the effects of matrine on CXCL12-induced cell migration activities in tumor cells. For this experiment, the cells were scarred with vertical and horizontal, then we observed the changing in width of wound following CXCL12-induced cell migration ability. In A549, DU145, and MIA PaCa-2 cells, CXCL12-induced cells had narrower width of wound because of more activation on cell migration. However, matrine significantly inhibited the cell migration compared with CXCL12-stimulated cells (Figure 2A–C).

### 2.4. Matrine Suppresses Invasive Potential of A549, DU145, and MIA PaCa-2 Cells

**Figure 3:**
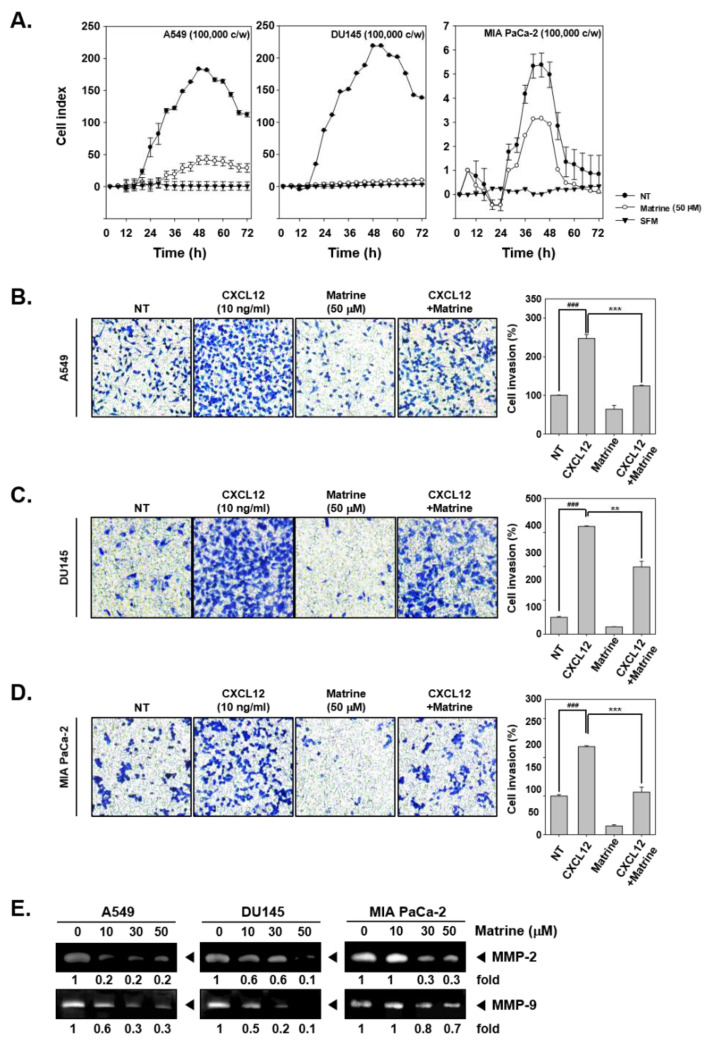
Matrine suppressed cell invasion in A549, DU145, and MIA PaCa-2 cells. (**A**) A549, DU145, and MIA PaCa-2 cells (1 × 10^5^ cells/well) were seeded on matrigel-coated CIM (cellular invasion/migration)-Plate 16 then treated with matrine (50 µM). Cell invasion activity was measured every 15 min intervals by Roche xCELLigence Real-Time CellAnalyzer (RTCA) DP instrument (Roche Diagnostics GmbH, Rotkreuz, Switzerland). (**B**–**D**) Membrane was coated with matrigel and dried for 1 h on room temperature. Membrane and chamber was combined, then cells were seeded on top chamber with CXCL12 (10 ng/mL) and matrine (50 µM). Chamber was incubated in 37 °C, 5% CO_2_ incubator 6 h for A549 cells, 4 h for DU145 cells, and 5 h for MIA PaCA-2 cells. (**E**) Zymography was examined for determined gelatinase activity. A549, DU145, and MIA PaCa-2 cells (5 × 10^5^ cells/well) were seeded on 6 well plate. Matrine was treated for 24 h and supernatant was obtained from each sample. Concentrated supernatant was prepared with equal amount of proteins. Then MMP-2 and MMP-9 gelatinase activity was compared on each cell lines. All experiments were performed independently at least 3 times and representative data are shown. ^###^ *p* < 0.001, vs. non-treated (NT) cells and ^***^ *p* < 0.001, ^**^ *p* < 0.01 vs. CXCL12 + matrine treated cells.

The potential ability of matrine to affect invasion activity was determined by real time cell analysis (RTCA) and boyden chamber assay. The results indicated that, matrine (50 µM) clearly suppressed cell invasion activity of A549, DU145, and MIA PaCa-2 cells in RTCA (Figure 3A). Cell invasion activity was also measured by boyden chamber assay with A549 (Figure 3B), DU145 (Figure 3C), and MIA PaCa-2 (Figure 3D). For this, cells were seeded on transwell chambers, and cell invasion was measured by cell translocation through matrigel-coated membrane. As shown in results, invaded cells were stained and observed in blue, matrine treated cells displayed lower invasion activity and stained less than CXCL12-induced cells. We then determined if matrine can also affect the levels of MMP-2 and MMP-9 proteins by gelatin zymography. A549, DU145, and MIA PaCa-2 cells were treated with matrine (0, 10, 30, 50 µM) for 24 h, thereafter concentrated supernatants were prepared for gelatin zymography. The expression of MMP-2 and MMP-9 was found to be drastically reduced in the treated cells (Figure 3E).

### 2.5. Matrine Does Not Affect the Degradation of CXCR4

**Figure 4:**
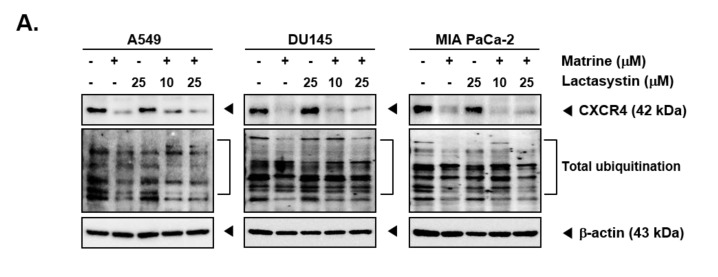

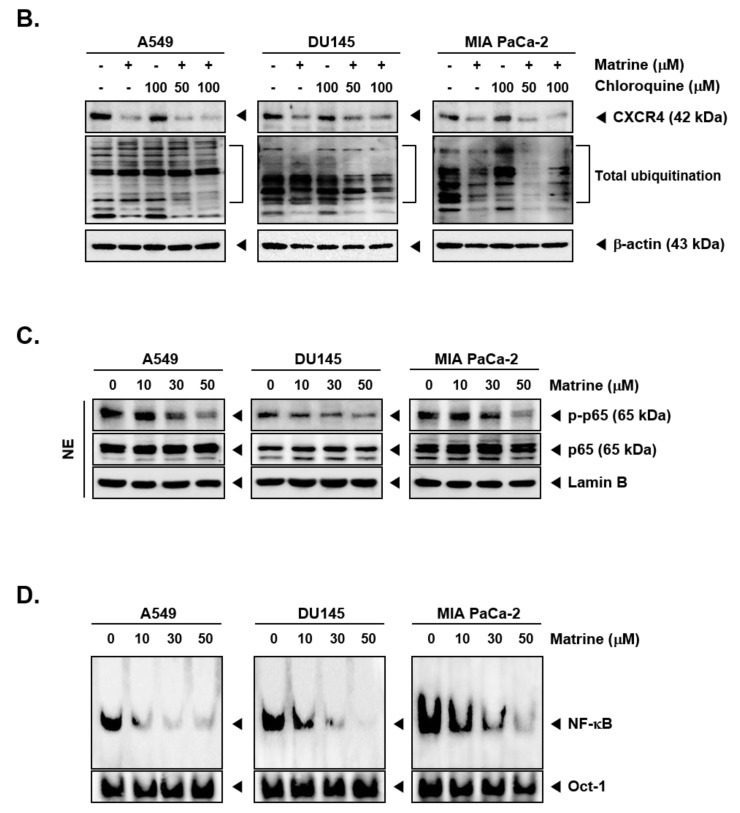
Matrine inhibits NF-κB activation in A549, DU145, and MIA PaCa-2 cells. A549, DU145, and MIA PaCa-2 cells (5 × 10^5^ cells/well) were pre-treated with (**A**) Lactasystin for 3 h or (**B**) Chloroquine for 3 h, then 50 µM of matrine treated for 24 h. Whole cell lysates were prepared for western blot analysis. Proteins were separated on SDS-PAGE gel and transferred on nitrocellulose membranes. Membranes were probed with anti-CXCR4 antibodies. (**C**) A549, DU145, and MIA PaCa-2 cells (5 × 10^5^ cells/well) were treated with matrine for 24 h. Nuclear extracts were extracted from cells and analyzed by western blot analysis. Proteins were probed with anti-phospho-p65 and anti-p65, then membranes were stripped and re-probed with anti-Lamin B antibodies. (**D**) A549, DU145, and MIA PaCa-2 cells (5 × 10^5^ cells/well) were treated with matrine for 24 h. Nuclear extracts were prepared to analyzed for NF-κB activation levels by Electrophoretic Mobility Shift Assay (EMSA). Oct-1 was observed as control. All experiments were performed independently at least 3 times and representative data are shown.

Matrine treatment may affect the expression of CXCR4 by promoting its degradation following total ubiquitination [56]. To investigate the mechanisms controlling the actions of matrine on CXCR4 levels, A549, DU145, and MIA PaCa-2 cells were pre-treated with proteasome blocker lactacystin (10 or 25 µM), for 3 h to abrogate CXCR4 degradation and thereafter treated with matrine (50 µM) for 24 h. As shown in Figure 4A, no substantial effect on matrine-induced CXCR4 downmodulation was observed in the presence of lactacystin (Figure 4A). Next, cells were also pre-treated with lysosomal inhibitor, chloroquine (50 or 100 µM), for 3 h and then treated with matrine (50 µM) for 24 h. As shown in Figure 4B, chloroquine could not also prevent the downregulation of CXCR4 in the same manner as observed with proteasome inhibitor.

### 2.6. Matrine Affects Phosphorylation of p65 in A549, DU145, and MIA PaCa-2 Cells

Because phosphorylation of p65 can regulate its transcriptional activity [57,58,59,60,61], we confirmed the effect of matrine on p65 phosphorylation inhibition (Figure 4C). A549, DU145, and MIA PaCa-2 cells were treated with matrine (0, 10, 30, 50 µM) for 24 h, then prepared the nuclear extracts for Western blot analysis. As shown in results, matrine clearly suppressed the phosphorylation of p65 on A549, DU145, and MIA PaCa-2 cells but there were no effects on p65 expression.

### 2.7. Matrine Mitigates Constitutive Activation of NF-κB in A549, DU145, and MIA PaCa-2 Cells

To determine the effects of matrine on constitutive activation of NF-κB, we employed EMSA in A549, DU145, and MIA PaCa-2 cells.

Cells were treated with matrine for 24 h and nuclear extracts were analyzed to observe the protein-DNA binding activity. The results suggested that matrine effectively blocked NF-κB activation in a dose-dependent manner (Figure 4D).

### 2.8. Matrine Inhibits Translocation of p65 in the Nuclei

**Figure 5:**
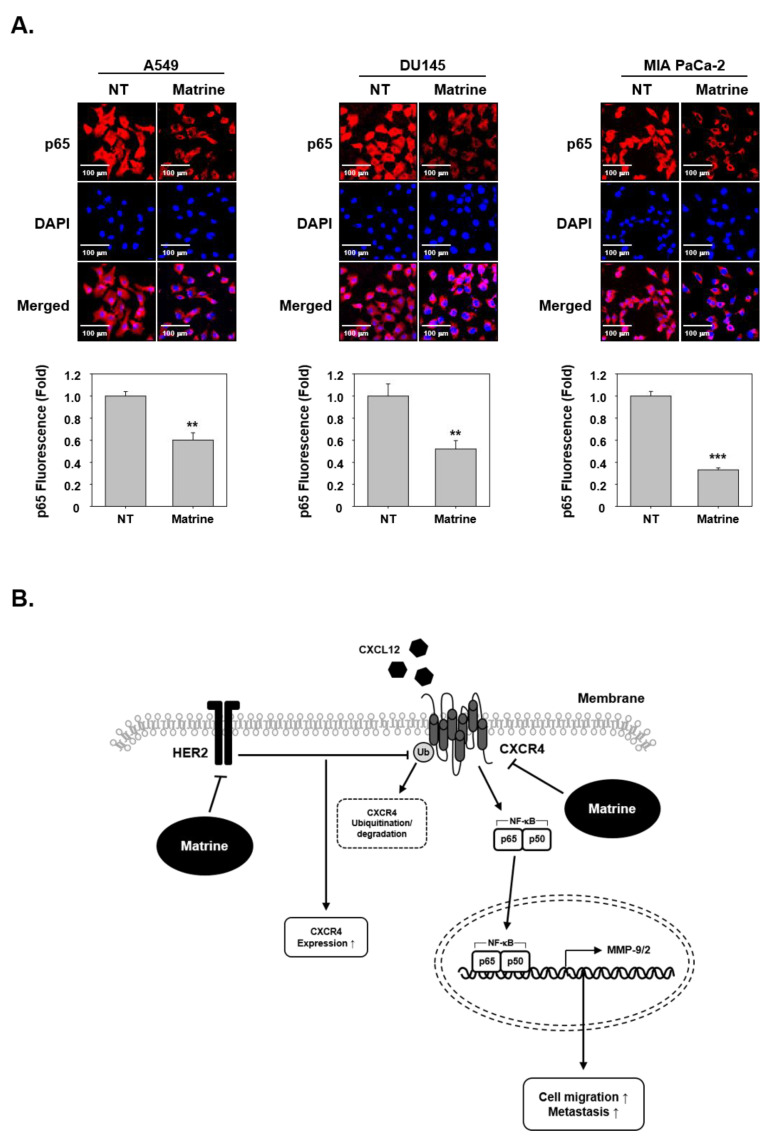
Matrine inhibits translocation of NF-κB into nucleus in A549, DU145, and MIA PaCa-2 cells. (**A**) p65 translocation into nucleus was observed by immunocytochemical analysis. A549, DU145, and MIA PaCa-2 cells (2 × 10^4^ cells/well) were treated with matrine (50 µM) for 24 h in 37 °C, 5% CO_2_ incubator. P65 was observed in red, nuclear was observed in blue color. Cells were analyzed by Olympus FluoView FV1000 confocal microscope (Tokyo, Japan). (**B**) A proposed model of the matrine effect on CXCR4 activation. All experiments were performed independently at least three times and representative data are shown. ^***^ *p* < 0.001, ^**^ *p* < 0.01 vs. non-treated (NT) cells.

To investigate whether matrine had effects on p65 translocation, we examined immunocytochemical analysis. The results indicated that matrine treated cells showed an inhibitory effect on the translocation of p65 into nuclei (Figure 5A).

## 3. Discussion

Our goal was to investigate the anti-cancer actions of matrine, especially its anti-metastasis activities with reference to the CXCR4 signaling axis in various cancer cells. In lung, prostate, and pancreatic cancer cells, we found that matrine displayed tolerable cytotoxicity at the various tested concentrations. It also significantly altered the levels of CXCR4 and MMPs in A549, DU145, and MIA PaCa-2 cells.

In various previous studies have been reported that metastasis can be regulated via CXCR4 and MMPs enzymes such as MMP-9 and MMP-2 [16,62,63,64]. The importance of CXCR4 in the process of cancer metastasis is evidenced by its overexpression in variety of tumors like small-cell lung carcinoma, prostate cancer, pancreatic cancer, breast cancer, gastric cancer, glioma, colon carcinoma, acute myeloid leukemia, ovarian cancer, melanoma, as compared to normal cells [19,24,65,66,67]. Our results suggest that the modulation of HER2 activity may influence the regulation of CXCR4 expression as HER2 receptor tyrosine kinase has been reported to play a role in inhibiting the ubiquitination of CXCR4 [25]. Therefore, we hypothesized that CXCR4 inhibition may occur at the transcriptional level. As expected, our results demonstrated that matrine can abolish the mRNA levels of CXCR4 and MMPs in A549, DU145, and MIA PaCa-2 cells.

CXCR4 can induce the activation of anti-apoptotic serine/threonine kinase, which can drive the MMPs secretion, activation, and expression to promote cellular invasion and migration [55]. MMPs, in addition to breaking down ECM, can affect the cell migration or invasion into blood, tissues, and other organs [68,69]. Hence, we confirmed that whether matrine can also affect the MMPs activation. Indeed, we found a substantial decrease of MMPs, especially MMP9 and MMP-2 activity, by matrine at both protein and mRNA levels in A549, DU145, and Mia PaCa-2 cells. In addition, we also observed the action of matrine on invasion of cancer cells and noted that matrine effectively abolished the invasive properties. Thus, our findings indicate that matrine may affect cancer invasion by down-regulation of MMP-9 and MMP-2 activities.

Up-regulation of CXCR4 has been related with activation of NF-κB, which can regulate tumor growth and survival [70,71]. As NF-κB binding site has been reported to be closely located within CXCR4 promoter and can modulate CXCR4 levels in breast cancer cells [72], we also investigated the impact of matrine NF-κB down regulation in this report. Our results suggested that it is possible to down-regulate the phosphorylation of NF-κB-p65 expression and translocation into nuclei upon matrine exposure in A549, DU145, and MIA PaCa-2 cells. In order to achieve p65 activation, it has to translocated into the nuclei, however, this process was blocked by matrine and as a result, p65 expression in the nuclei was significantly reduced.

Nowadays, lung, prostate and pancreatic cancer are considered as major cancers [2] and account for a large proportion of mortality. While the cancer mortality rate is decreasing, the deaths caused by lung cancer still remain signifcinatly high, thereby highlighting the need for new treatment options. Our study demonstrates that matrine can effectively down-regulate the cellular invasion and metastasis through substantially affecting CXCR4, MMP-9, MMP-2, and NF-κB levels (Figure 5B). Therefore, matrine may be used as anti-cancer agent that can exhibit potential against metastatic ability of tumor cells.

## 4. Materials and Methods

### 4.1. Reagents

Matrine (Figure 1A) was supplied by Narula Research (Chapel Hill, NC, USA) and dissolved in 1× PBS then stored in 100 mM stock solution at −20 °C. Tris base, glycine, NaCl, sodium dodecylsulfate (SDS), 3-(4,5-dimethylthiazol-2-yl)-2,5-diphenyltetrazolium bromide (MTT), and bovine serum albumin (BSA) were also purchased from Sigma-Aldrich (St. Louis, MO, USA).

### 4.2. Cell Lines and Culture Conditions

Human non-small cell lung carcinoma (NSCLS) cells A549, human prostate cancer cells DU145, and human pancreas cancer cells MIA PaCa-2 was obtained from American Type Culture Collection (Manassas, VA, USA). A549 cells were cultured in DMEM-low medium containing 10% inactivated-FBS and 1% penicillin-streptomycin. DU145 and MIA PaCa-2 cells were cultured in RPMI-1640 medium containing 10% inactivated-FBS and 1% penicillin-streptomycin.

### 4.3. MTT Assay

To measure the cell viability for matrine treatment, MTT assay was examined. A549, DU145, and MIA PaCa-2 cells (1 × 10^4^ cells/well) were seeded on 96 well plate for overnight in 37 °C, 5% CO_2_ conditions. All cells were treated with matrine (0, 10, 30, 50 μM) for 24 h, matrine and oxymatrine were diluted in the cultured media. After 24 h, 30 μL of MTT solution (2 mg/mL) was added on each wells and incubated at 37 °C in 5% CO_2_ conditions for 2 h. Then to dissolve the MTT formazan, 100 μL of MTT lysis buffer was added for overnight at 37 °C incubator. Cell viability was measured by VARIOSKAN LUX device (Thermo Fisher Scientific Inc, Waltham, MA, USA) at 570 nm [73].

### 4.4. Western Blot Analysis

Cells (5 × 10^5^ cells/well) were seeded on six-well platex and incubated for overnight in 37 °C, 5% CO_2_ conditions. Cells were treated with various indicated concentrations and time intervals then cells were lysed and total concentrations of protein was quantified through a Bradford assay (Bio-Rad, Hercules, CA, USA). Proteins were prepared with equal amounts (10 μg) for western blot, then proteins were separated on SDS-PAGE gels to each protein size. Separated proteins were transferred on nitro cellulose membranes and blocked with 5% skim milk solution for 1 h at room temperature. Membranes were washed with 1×TBST (1×TBS with 0.1% Tween 20) and probed with primary antibodies at 4 °C for overnight. Membranes were washed with 1×TBST then incubated with secondary antibodies at room temperature for 1 h. After membranes were washed again, detected by enhanced chemiluminescence (ECL) kit (EZ-Western Lumi Femto, DOGEN, Guro, Korea) [74].

### 4.5. RT-PCR

To evaluate the effects of matrine (50 μM, 0-6-12-24 h) on mRNA levels, total RNA was extracted from each cells. RNA was prepared of 1 g for each cDNA premix kit (Maxime RT PreMix, Intron, Dasjeon, Korea) to convert to cDNA. Then RT-PCR was performed for CXCR4, MMP-2, and MMP-9. CXCR4 was performed at 94 °C for 5 min, 94 °C for 30 s, 50 °C for 30 s, 72 °C for 30 s with 30 cycles and extension at 72 °C for 7 min. MMP-2 was performed at 95 °C for 5 min, 95 °C for 1 min, 62 °C for 1 min, 72 °C for 4 min with 30 cycles. MMP-9 was performed at 94 °C for 5 min, 94 °C for 30 s, 62 °C for 30 s, 72 °C for 30 s with 30 cycles and extension at 72 °C for 7 min. mRNA was resolved on 1% agarose gel and determined expression of mRNA levels. Glyceraldehyde-3-phosphate dehydrogenase (GAPDH) was used as loading control [75].

### 4.6. Wound Healing Assay for Cell Migration Observation

Cell migration change by matrine treatment was observed by wound healing assay. A549, DU145, and MIA PaCa-2 cells (5 × 10^5^ cells/well) were seeded on 6 well plate. Cells were scarred with vertical and horizontal lines and replaced fresh serum-free medium, then treated with CXCL12 (10 ng/mL) or matrine (50 μM) for 24 h. Width of wounds was monitored at 0 h and 24 h, measured microscopically by observed by using a microscope (ECLIPSE Ts2, Nikon, Tokyo, Japan) [76].

### 4.7. Invasion Assay by RTCA

Invasion activity of cells were determined by xCELLigence Real-Time Cell Analyzer (RTCA) DP instrument (Roche Diagnostics GmbH, Basel, Switzerland). Top chamber (invasion/migration (CIM)-Plate 16) was pre-coated with matrigel (BD Biosciences, Becton-Dickinson, Franklin Lakes, NJ, USA) for 4 h, add only 10% FBS containing medium to under chamber then chambers were assembled. Serum-free medium was previously measured then A549, DU145, and MIA PaCa-2 cells were seeded on top chamber with matrine (50 μM). Cell index was measured every 15 min time intervals [77].

### 4.8. Boyden Chamber Assay for Cell Invasion Observation

To determine the effect of matrine on invasion in vitro, we performed a Boyden chamber assay. Matrigel-coated 8-μm polycarbonate membrane was prepared on transwell chamber. A549, DU145, and MIA PaCa-2 cells (2 × 10^4^ cells/well) were seeded on top chamber with matrine (50 μM) or CXCL12 (10 ng/mL) in medium then incubated at 37 °C in 5% CO_2_ conditions for 5 h. After incubation, polycarbonate membrane was fixed and stained with Diff-Quick staining kit. Then cell invasion was observed by using a microscope (Nikon ECLIPSE Ts2) [78].

### 4.9. Gelatin Zymography

Gelatinolytic activity of MMP-2 and MMP-9 was evaluated by gelatin zymography. A549, DU145, and MIA PaCa-2 (5 × 10^5^ cells/well) cells were treated with matrine (0, 10, 30, 50 μM) for 24 h. The supernatants were concentrated and prepared equal amounts for gelatin zymography. Samples were separated on 0.1% gelatin contained 10% SDS-PAGE gel. Gels were washed by 2.5% triton X-100 for 1 h and incubated in zymo-reaction buffer at 37 °C, 5% CO_2_ conditions for overnight. Next, gels were stained with coomassie brilliant blue (7% glacial acetic acid, 40% methanol, 0.25% Coomassie Brilliant Blue R250) then destained with destaining buffer (10% glacial acetic acid, 10% methanol) until band was observed [76].

### 4.10. EMSA

To evaluate the NF-κB-DNA binding activation, we confirmed the expression of NF-κB level with nuclear extracts of A549, DU145, and MIA PaCa-2 cells by EMSA. The cells (5 × 10^5^ cells/well) were seeded on 6 well plate and treated with matrine (0, 10, 30, 50 μM) for 24 h. Cells were harvested and nuclear extracts were prepared for equal amounts (5 μg), then incubated with NF-κB oligonucleotide probe (5′-AGTTGAGGGGACTTTCCCAGGC-3′ and 5′-GCCTGGAAAGTCCCCT CAACT-3′) in room temperature for 20 min. Oct-1 (5′-TTCTAGTGATTTGCATTCGACA-3′ and 5′-TGTCGAATGCAAATCACTAGAA-3′) was used as loading control. DNA-protein complex was resolved in 5% native polyacrylamid gels and transferred to nylon membrans. Then membranes were reacted by LightShift^®^ Chemiluminescent EMSA kit (Thermo Fisher Scientific Inc., Waltham, MA, USA) [79,80].

### 4.11. Immunocytochemistry

The translocation of NF-κB into nucleus was observed by immunocytochemical analysis. A549, DU145, and MIA PaCa-2 cells (2 × 10^4^ cells/well) were seeded and treated with matrine (50 μM) for 24 h. Cells were fixed with 4% paraformaldehyde for 20 min, incubated with 0.2% Triton X-100 for 10 min at room temperature. Cells were washed by PBS and blocked by 5% BSA in PBS for 1 h, then probed with anti-p65 antibody at 1:100 dilution in 4 °C for overnight incubation. After overnight incubation, cells were incubated with donkey anti-rabbit IgG-Alexa 594 (Molecular Probes, Carlsbad, CA, USA) at 1:1000 dilution for 1 h at room temperature. Cells were washed by PBS, and stained nuclear with DAPI for 3 min. Then cells were mounting with mounting medium from Sigma-Aldrich and detected by fluorescence microscopy (FluoView FV1000 confocal microscope, Olympus, Tokyo, Japan) [81].

### 4.12. Statistical Analysis

All numeric values are represented as the mean ±  SD. Statistical significance of the data compared with the untreated control was determined using the Student unpaired *t*-test. Significance was set at * *p*  <  0.05, ** *p*  <  0.01, and *** *p*  <  0.001.

## Abbreviations

CXCR4	C-X-C chemokine receptor type 4
NF-κB	Nuclear factor κB
CXCL12	C-X-C motif chemokine 12
HER2	Human epidermal growth factor receptor 2
VEGF	Vascular endothelial growth factor
MMP	Matrix metalloproteinases
GAPDH	Glyceraldehyde 3-phosphate dehydrogenase
TGFβ	Transforming growth factor beta
SDS	Sodium dodecyl sulfate
PBS	Phosphate buffered saline
TBS	Tris buffered saline
ECL	Enhanced chemiluminescence
FBS	Fetal bovine serum
MTT	3-(4,5-dimethylthiazol-2-yl)-2,5-diphenyltetrazolium bromide
